# Bridging the research–practice gap in infant neuromotor development by harnessing principles from implementation science

**DOI:** 10.3389/fresc.2026.1985679

**Published:** 2026-10-28

**Authors:** Samuel Carey, Elizabeth Hoffman, Cheryl Patrick, Jessica Hunter, Sarah Babula, Eleanor Clark, Megan K. O’Brien, Nicole Pouppirt, Raye-Ann deRegnier, Richard L. Lieber, Arun Jayaraman

**Affiliations:** 1 Shirley Ryan AbilityLab, Chicago, IL, United States; 2 Division of Rehabilitative Services, Ann & Robert H. Lurie Children’s Hospital of Chicago, Chicago, IL, United States; 3 Pathways.org, Chicago, IL, United States; 4 Department of Physical Medicine & Rehabilitation, Feinberg School of Medicine, Northwestern Medicine, Chicago, IL, United States, United States; 5 Division of Neonatology, Ann & Robert H. Lurie Children’s Hospital of Chicago, Chicago, IL, United States; 6 Department of Pediatrics (Neonatology), Feinberg School of Medicine, Northwestern University, Chicago, IL, United States; 7 Department of Biomedical Engineering, Querrey Simpson Institute for Bioelectronics, Northwestern University, Evanston, IL, United States; 8 Department of Neuroscience, Feinberg School of Medicine, Northwestern University, Chicago, IL, United States; 9 Edward Hines V.A. Medical Center, Hines, IL, United States; 10 Department of Physical Therapy and Human Movement Sciences, Feinberg School of Medicine, Northwestern University, Chicago, IL, United States; 11 Department of Medical Social Sciences, Feinberg School of Medicine, Northwestern University, Chicago, IL, United States

**Keywords:** clinical theory, clinical trial design, implementation science, neuromotor development, pediatric rehabilitation, resource development

## Abstract

The well-documented research–practice gap in healthcare typically results in an average delay of 17 years between evidence discovery and clinical care integration. This perspective article describes the application of implementation science methods to operationalize new clinical-research concepts into a scalable early physical therapy intervention supporting infant neuromotor development. Through flexible dosage delivery, adaptation of existing resources, structured stakeholder review, standardized training protocols, and systematic fidelity monitoring, we created this intervention for efficient dissemination and uptake. Using a case example, we also demonstrate effective implementation of our clinical-research concepts and suggest that the use of these specific processes of embedding implementation science tenets within trial design may help narrow the research–practice gap across disciplines.

## 1 Introduction

A persistent challenge across many clinical disciplines is aligning evidence-based theory with practice to improve real-world diagnosis and decision-making. This well-documented research–practice gap can lead to misdiagnosis, lack of treatment, or ineffective care (1). The uptake of new evidence into routine clinical practice takes an average of 17 years (2), and nearly 70% of change efforts fall short of full implementation (3). This gap is particularly consequential in the field of child development where early intervention is crucial (4). With one in six children in the United States at risk for developmental disabilities (5), there is a critical need to accelerate this pipeline. Delayed implementation not only slows delivery of effective interventions but risks widening disparities in early identification, access to care, and long-term developmental outcomes (6). Improving this process is not only a matter of translational efficiency but a public health necessity with implications for population-level child development and wellbeing.

To improve the efficiency of clinical adoption without compromising scientific rigor, principles from implementation science must be systematically applied. Implementation science examines methods that promote the uptake of evidence-based practice into routine clinical care. Research demonstrates that evidence is more likely to be translated into practice when structured implementation strategies are used (7). These include establishing an implementation committee to guide planning, decision-making, and accountability; adapting and tailoring theoretical frameworks to fit the local context in which the intervention is delivered; and providing ongoing clinician support through training, feedback, and facilitation (8, 9). Together, these strategies help bridge the gap between evidence and practice by addressing contextual barriers, enhancing stakeholder engagement, and supporting sustained behavioral change.

Here, we demonstrate an example of the use of implementation science tenets to design and test a clinical trial by focusing on dosage delivery, resource development, clinician training, and fidelity monitoring. This trial is currently ongoing as the Project CORB–Northwestern–Shirley Ryan AbilityLab–Lurie Children's-Pathways.org Infant Early Detection, Intervention and Prevention Project (Project CORB) (Lurie Children's Hospital Institutional Review Board #2022-5243, Northwestern University IRB #STU00217496, University of Illinois Chicago IRB#STUDY2022-0945). Project CORB is a multisite longitudinal randomized controlled trial initiated in the neonatal intensive care unit (NICU) and continued to 12 months’ corrected age (CA) (10). The goal is to create an evidence-based and easily scalable early physical therapy (PT) intervention for infants at risk for neuromotor delay. We posit that prioritizing these elements in study design will streamline integration into future clinical practice, providing an effective approach to narrowing the research–practice gap in early childhood development.

## 2 Translating theoretical framework into clinical trial design

The initial phase of Project CORB used an extensive literature review, expert consensus, and stakeholder feedback to establish best practices for optimizing infant neuromotor health during the first year of life. The resulting framework was summarized as having four major components: Caregiver engagement, Active leaRning, Environmental enrichment, and Strengths-based (CARES) (11). The CARES framework is a transdiagnostic approach that positions caregivers as equal partners in their child's care and emphasizes infant-directed action as the primary mechanism of change. As a relational-health model, it departs from traditional impairment–focused, clinician-directed care by utilizing strengths-based, activity-dependent learning within everyday caregiving contexts to optimize development during periods of heightened neuroplasticity.

As the CARES framework was being adapted into a clinical trial design, the Project CORB study team added a new principle: high dosage (10). Dosage refers to the frequency and duration of therapy sessions and the time infants actively participate in activities targeting weekly motor goals. This principle was selected for Project CORB to capitalize on heightened neuroplasticity during early infancy (12, 13) and align with evidence linking early intervention dosage with functional outcomes (14). The study team selected a high-dosage therapeutic intervention of two sessions per week, with each session lasting 60 minutes. This is notably higher than what an infant typically receives through standard-of-care early intervention in the first year, which often begins sometime after 3-months’ corrected age and averages 45 min weekly (14–16). In addition, caregivers contribute to therapy dosage through daily activities, supported by positive infant motor outcomes from a caregiver-led PT intervention (17).

Although the CARES framework and high-dosage principles provide guidance on how to deliver early neuromotor interventions, they offer limited direction for operationalizing these concepts within clinical contexts for eventual real-world application. In particular, they do not offer clear parameters for delivery, feasibility, or adherence support. To address this, the Project CORB team developed the following strategies informed by established tenets of implementation science, with the goal of optimizing intervention delivery, while maintaining feasibility, flexibility, and family empowerment.

### 2.1 Creating high-dosage feasibility

Although high-dosage therapy is frequently recommended across rehabilitative conditions, adherence is often limited by practical barriers. Session frequency and duration, transportation time and cost, competing work demands, and physical fatigue may contribute to missed visits and attrition (18). These challenges are particularly pronounced for this participant group, as infant care places substantial time demands on families (19). To increase adherence, implementation science suggests using strategies to reduce logistical burden and incorporate dosage into daily life (20).

For PT sessions in Project CORB, a flexible scheduling approach was used to reduce competing time demands on families. The primary mode of session delivery was in-person, either at NICU bedside or within the family's home. Telehealth sessions were offered as a secondary option so that services may continue instead of cancelation. Previous studies demonstrated that telehealth is a viable alternative mode of early intervention therapies (21). This approach reduced travel-related burden and allowed intervention sessions to proceed during periods of participant or caregiver illness or other unforeseen circumstances, minimizing missed visits and supporting intervention adherence.

For caregiver-led home activities, caregivers were encouraged to work activities into the infant's daily routine in small intervals of time, as an alternative to blocked practice (15). Activities were also divided into caregiver-led and infant-led with a structured environment to maximize opportunities for active learning throughout the day.

### 2.2 Developing existing resources

A substantial set of supporting resources were needed to implement the CARES framework effectively at high dosage. To align with our goal of narrowing the research–practice gap, we recognized that Project CORB's early PT intervention must be practical and scalable. Resources were required to align with active learning principles, use accessible objects, and be feasible within daily routines. Because research suggests that using multiple modes of educational materials (written, visual, auditory, and demonstration) best supports caregiver learning with their child (22), we also needed resources to accommodate different learning styles.

Rather than developing materials in a ground-up approach, thereby prolonging implementation, we partnered with Pathways.org to leverage their extensive resources. Over the last 40 years, Pathways.org created structured play activities, termed “games,” with guidance from expert pediatric clinicians (*n* = 45; PT, Occupational Therapy (OT), Speech Language Pathology (SLP)) and developmental specialists (23). These activities are presented online through short videos designed to show families how to support milestone achievement at different developmental stages.

To adapt the games of Pathways.org to meet the requirements of Project CORB, we needed to first establish developmental goals. Through expert clinician review (*n* = 3), we established developmental “blocks” associated with distinct therapeutic goals. Instead of milestone-based achievements, Project CORB's goals focused on tasks and functional movements that promoted maximizing the infant's independence in their natural environment. Next, we established parameters to ensure that intervention activities provide “just-right challenges” to maximize active learning. This was achieved by adjusting the environment and structuring the activity so that the infant requires minimal support to be successful (11). The appropriate environmental enrichment strategy was selected through physical therapist and caregiver collaboration (24). Activity challenge level was individualized for each participant by using the “wait, show, help, stop” concept (11), which prioritizes self-initiated movement and guides the therapist and caregiver through activity scaffolding. We excluded any standard-of-care PT interventions that restricted infant-directed movement or did not support active learning. Excluded interventions included orthotics and adaptive equipment; strictly passive interventions (e.g., range of motion, muscle elongation/stretching, massage, and kinesiotaping); and positioning aides (e.g., blanket rolls or bolsters) in prone.

Once intervention goals and activity parameters were established, a rigorous video grading process took place to ensure that the selected Pathways.org games fit within the evidence-based framework. A panel of physical therapists (*n* = 2) and an instructional designer (*n* = 1) reviewed the games and associated video clips for alignment with key principles and goals. Any game not focused on gross motor development was excluded. Games misalignment with one or more key principles or poor video quality was marked for revision or exclusion. New videos were recorded to address identified educational gaps. In total, 683 video clips were rigorously reviewed; 559 video clips were original to Pathways.org and 124 clips were newly recorded by the study team (Figure 1). In addition, existing caregiver education materials from the Ann and Robert H. Lurie Children's Hospital of Chicago Pediatric PT Department were reviewed. These included NICU handouts related to behavioral state regulation, reading infant cues, calming strategies, and alerting strategies. All content was revised to align with the intervention framework.

**Figure 1:**
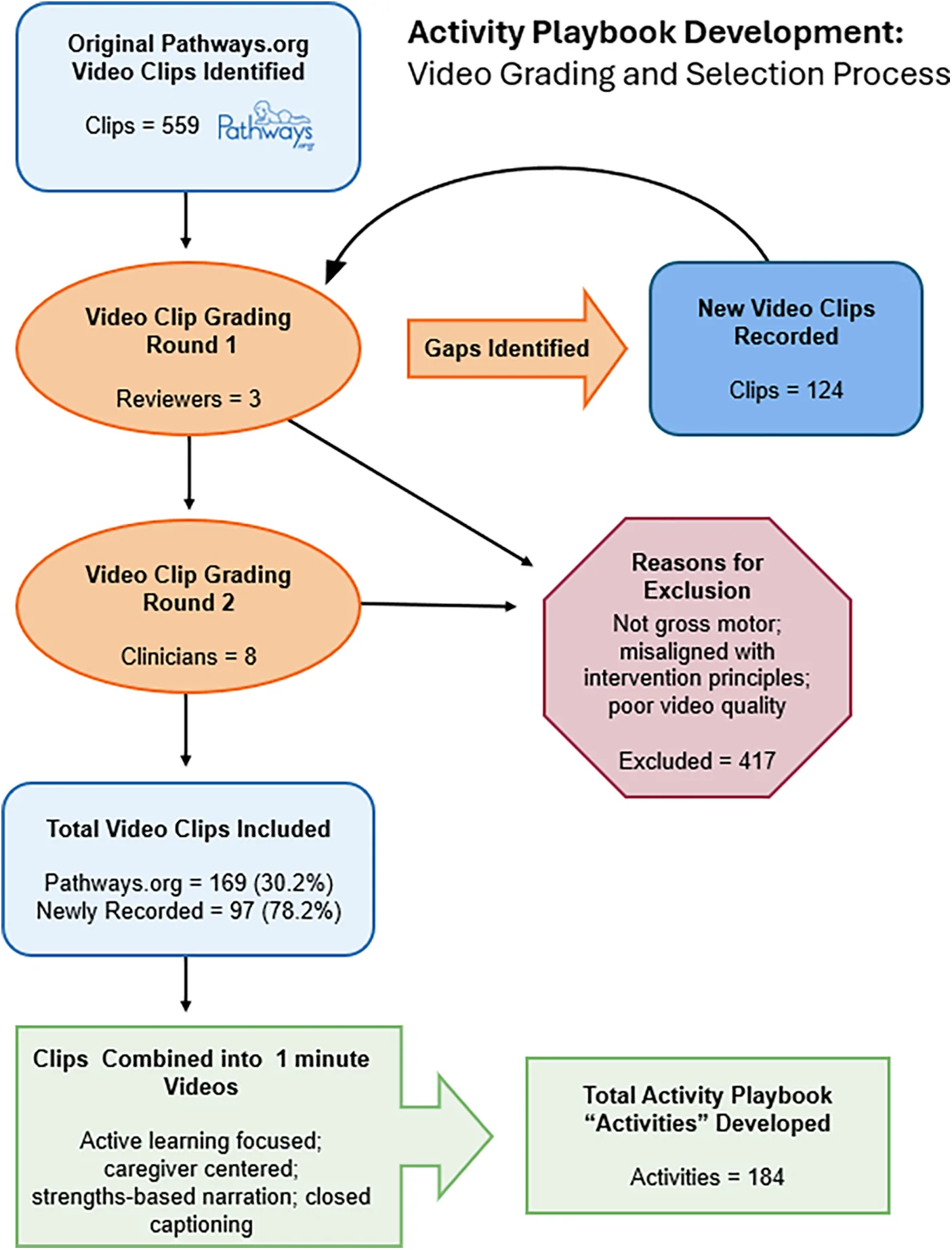
Activity Playbook development. The video clip grading process (for existing Pathways.org videos and newly recorded videos) and pipeline to establish “activity” videos for the Activity Playbook used in caregiver education during the Project CORB Intervention.

After an initial review, all materials were evaluated by a panel of pediatric physical therapists (*n* = 8) with an average of 25 years of experience working in the NICU and with infants at risk for neuromotor delays. They provided feedback on the selected video clips, final revisions, and on enhancing the user experience and accessibility of educational materials. They also offered recommendations for supplemental materials to complement videos and support different learning styles.

After all reviews were complete, 266 video clips were included in the Project CORB Intervention (Figure 1). The final content was renamed “activities” and compiled into the Activity Playbook. Video clips were combined into Activity Playbook videos up to 1 min in length and included written text, narration, closed captioning, and depictions of caregivers and babies participating in activities. Language was standardized in all videos to reflect the key principles and emphasize a strengths-based approach. The Activity Playbook now includes a total of 184 videos that span the spectrum of development from birth to 1 year and are tailored for both the NICU and home environments. Videos are inclusive of all ability levels and depict ways to scaffold activities through caregiver engagement and environmental enrichment.

Multiple modes of caregiver educational materials were created to support different learning styles, as both written and video materials are known to be effective educational tools in early intervention therapy programs (25). Caregivers received the Activity Playbook as an educational resource during the intervention. Activity Playbook cards were created for every video to provide caregivers with a quick visual reminder of assigned Activities. Based on NICU clinician feedback, premade educational handouts were used during the initial NICU stay.

During the intervention, caregivers and therapists participate in joint weekly goal-setting conversations. Collaborative planning improves adherence to recommended home activity programs in early intervention programs (15, 25). Goals were short term and designed to promote active learning, provide an appropriate challenge based on the infant's readiness, and prioritize the caregiver's preferences. Up to three Activity Playbook videos were selected by the physical therapist in collaboration with the caregiver to support weekly goals. Collectively, this review process shortened the timeline for developing evidence-based resources that aligned with the CARES framework, without compromising rigor, caregiver acceptability, or ease of implementation.

### 2.3 Maintaining intervention rigor: clinician training and fidelity monitoring

After resource development and establishing intervention procedures, we implemented a training protocol enabling clinician uptake and adherence. Clinician buy-in is a critical, often decisive, factor in the success of new practice recommendations. Without active support and an understanding of the underlying theory, even well-funded or technologically advanced initiatives frequently fail, as clinicians often function as gatekeepers for adoption, particularly at the point of care (26).

Study team members (*n* = 5) initially wrote two manuals establishing a standardized training process for therapists providing the intervention. These were divided by implementation setting and included a NICU Therapist Training Manual (for enrollment to NICU discharge) and a Home Therapist Training Manual (for NICU discharge until 12-months’ CA). Both manuals included background information, key principle definitions, documentation requirements, therapy session guides, and explanation of goals and the Activity Playbook. Content experts (*n* = 5) provided feedback on structure, wording, and Activity Playbook implementation. The manual was then brought to clinicians (*n* = 3), who provided feedback on the fully assembled manuals.

Next, a training program was created. Physical therapists were taught the theoretical framework for the intervention and oriented to the Activity Playbook. All physical therapists were required to pass a written examination to establish implementation reliability. A challenge to therapy implementation is long-term adherence. Therefore, we incorporated fidelity monitoring to ensure consistent intervention delivery (27). Every month, study physical therapists participated in fidelity monitoring of intervention sessions to grade adherence to the key principles. The sessions were video-recorded and scored by at least two independent reviewers. If the session fell below 85% adherence on any of the key principles, the therapist was retrained and reevaluated. This monthly review creates regular accountability, while video recording allows for objective, reproducible assessment rather than reliance on self-report. Scoring by at least two independent reviewers improves consistency and reliability, increasing confidence in fidelity ratings compared with single-rater designs. The retraining and reevaluation process ensures that deviations are corrected promptly. Together, this process promotes intervention integrity and enhances both internal validity and the likelihood of successful, scalable implementation.

## 3 Intervention case example

To illustrate the feasibility and adherence achieved through our implementation science-supported framework, we present a case example from a randomly selected participant of Project CORB. The data of this participant will be excluded from Project CORB's overall results.

### 3.1 Intervention implementation

The participant was a female infant born at 34 weeks of gestation, weighing 1,790 g, and identified with risk factors for neuromotor delay including left grade III intraventricular hemorrhage, left frontal cystic periventricular leukomalacia, and mild-to-moderate dilation of the left lateral ventricle. After informed consent, this participant was enrolled into Project CORB Intervention at 37 weeks’ postmenstrual age and randomized to the experimental intervention group. She had one study visit in the NICU prior to discharge home. Home-based sessions began within 9 days of discharge.

As previously outlined, the study intervention consisted of twice weekly tailored PT sessions focused on supporting caregivers’ development in problem-solving skills, recognizing infant stress and self-regulation cues, and fostering active learning and self-efficacy in the infant. In addition to receiving the intervention, the infant's neuromotor development was tracked throughout the study at enrollment, 1-, 2-, 3-, 6-, 9-, and 12-months’ CA utilizing a multidimensional evaluation paradigm including standardized clinical assessments and high-resolution inertial sensors. Pediatric Rehabilitation Intervention Measure of Engagement—Parent Version (PRIME-P) was administered monthly to assess the caregiver's perspectives on participating in the study intervention. Multiple assessments were used to evaluate intervention effectiveness (Figure 2). The test of infant motor performance assesses postural and selective movement control in young infants (28, 29). The Hammersmith Infant Neurological Examination (HINE) evaluates neurological function, including global and asymmetry scores (30–32). The Neurological Sensory Motor Developmental Assessment (NSMDA) assesses motor and neurological performance (33, 34), while the Alberta Infant Motor Scale (AIMS) measures gross motor development (35). The Bayley Scales of Infant and Toddler Development, Fourth Edition (Bayley-4), assesses developmental outcomes, including motor function (36).

**Figure 2:**
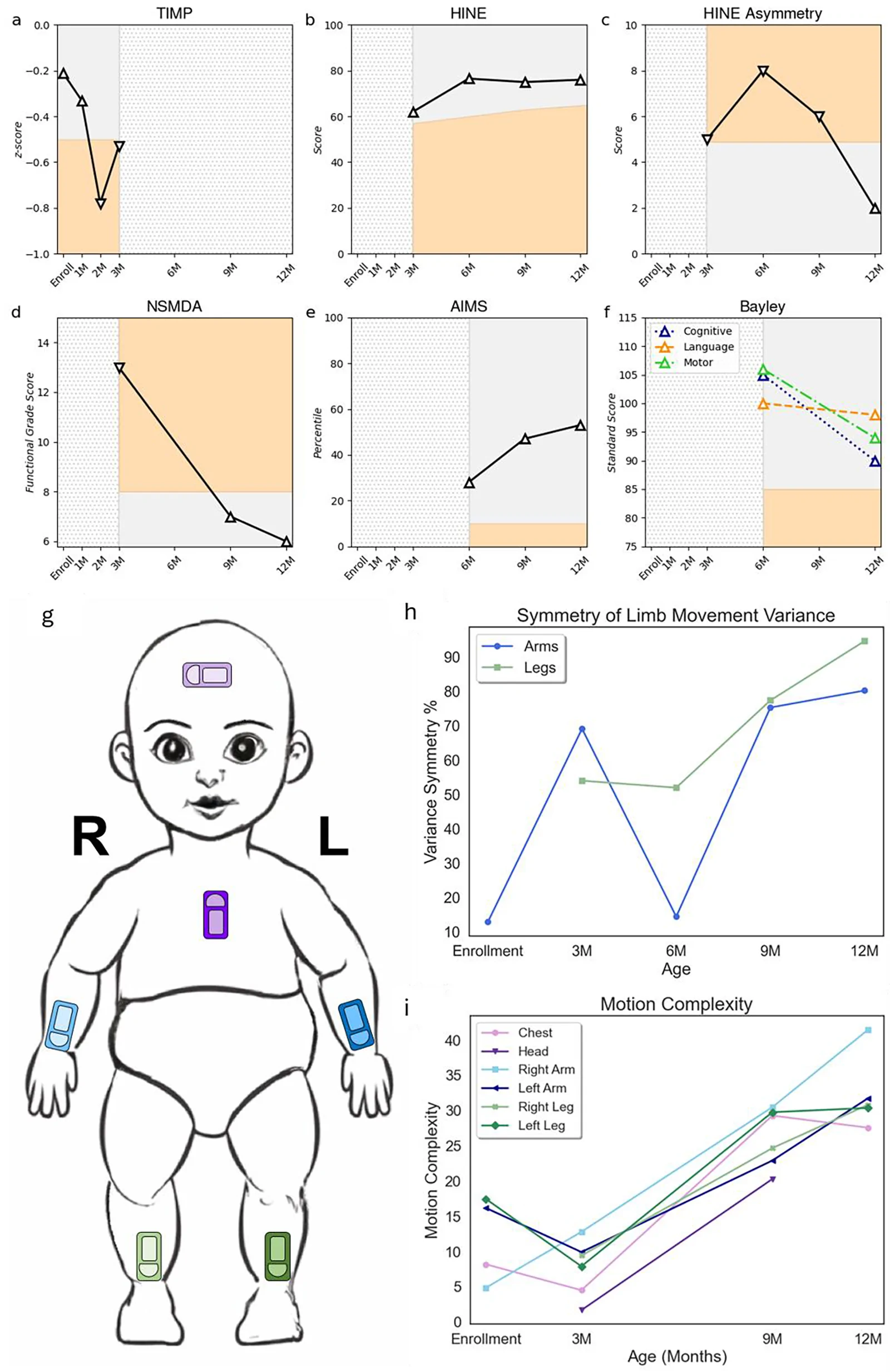
Case example outcomes. Standardized clinical assessment results denoted in **(a–f)**, where the shaded orange areas represent age-referenced score ranges classified as atypical according to established criteria of each assessment. Assessments include: **(a)** Test of Infant Motor Performance, **(b)** Hammersmith Infant Neurologic Examination (HINE) global score, **(c)** HINE asymmetry score, **(d)** Neurological Sensory Motor Developmental Assessment, **(e)** Alberta Infant Motor Scale, and **(f)** Bayley Scales of Infant and Toddler Development (4th edition). Wearable sensor information is represented in **(g–i)**, including: **(g)** a sensor placement diagram, **(h)** a symmetry of limb movement variance, with a value of 100% indicating equal variance of limb movements on each side, and **(i)** a motion complexity of each body segment, with higher values indicating more complex angular velocity movements.

### 3.2 Participation and outcomes

This participant averaged 1.90 PT intervention sessions per week over the course of 51.7 weeks. The majority of sessions occurred in person at the participant's home [*n* = 93, 94.9%; NICU (*n* = 1, 1.0%) and virtually (*n* = 4, 4.1%)]. Four caregivers engaged in the intervention and logged a total of 34,860 home activity minutes (averaging 674.7 ± 292.5 min per week). Seventy-three unique Activity Playbook videos were assigned over the course of the study, which guided caregivers in completing home-activity minutes. Activity Playbook videos were chosen to support weekly goals and individualized to the participant via coaching provided by the physical therapist. Seven fidelity monitoring sessions were reviewed and demonstrated an average of 96.3% ± 2.6% adherence to Project CORB Intervention principles.

Overall, this participant's motor function improved from 3 to 12 months’ CA (Figure 2). At 3, 6, and 9 months, a subset of assessments (General Movements Assessment Motor Optimality Score-Revised, HINE asymmetry, and high-resolution inertial sensors) indicated potential neuromotor delay, with limb asymmetry as a key concern. By 12 months’ CA, the participant showed no clinically significant limb asymmetry and performed within age-expected limits on all administered assessments. The caregivers viewed study participation favorably, as measured by PRIME-P. They reported high engagement with the intervention, hope and optimism about their child's progress, perceived appropriateness of the plan for their child's needs, and a collaborative relationship with the physical therapist. Although time commitment, fatigue, illness, and scheduling acted as barriers at times, caregivers found that scheduling flexibility, additional caregiver support, coaching from the physical therapist, and motivating Activity Playbook assignments supported their participation.

## 4 Discussion

Our method of supporting high-dosage feasibility, adapting existing resources, providing standardized training, and requiring ongoing fidelity monitoring during implementation may help close the research–practice gap for our intervention. A focus group of caregivers (*n* = 7) participating in Project CORB reported positive feedback around intervention feasibility and educational resources, findings further supported by the case example. Caregiver satisfaction with, and the feasibility of implementing, the intervention is further supported by the results of the case example.

As the trial is ongoing (final assessment, December 2026), the overall results of Project CORB are unknown. If there is efficacy, we have high confidence that this intervention can be smoothly implemented into clinical practice by trained clinicians and caregivers. Utilizing implementation science principles during trial design has prepared the study team for future widespread dissemination. Currently, Pathways.org offers their games, the source of our study's Activity Playbook videos, free for public use on their website and mobile application.

Our framework of high-dosage feasibility, resource development, clinician training, and fidelity monitoring offers a replicable approach to guide implementation efforts and reduce the research–practice gap. When possible, researchers should plan dosage support, adapt existing resources to align with novel therapeutic approaches, and establish rigorous training and fidelity review processes to ensure reliability. Adapting resources may require collaboration with outside organizations; we encourage researchers to seek out partnerships if applicable. This work demonstrates an effective method for advancing evidence-based research theory into a practical, implementable, intervention.

This report focused on intervention effectiveness and family-level implementation. Future research plans to evaluate system-level implementation factors, including training, staffing, costs, sustainability, and translation to practice. Previously published work further details our intervention descriptions, considerations for implementation, and preexisting approaches (10).

## Data Availability

The datasets presented in this article are not readily available because the data obtained during the course of this study may be available to non-profit third parties, subject to approval from the study Advisory Council. Only limited datasets or deidentified data will be available. The complete dataset will not be made publicly available as it contains private health information. Requests to access the datasets should be directed to Arun Jayaraman, ajayaraman@sralab.org.
